# Exogenously Applied Plant Growth Regulators Enhance the Morpho-Physiological Growth and Yield of Rice under High Temperature

**DOI:** 10.3389/fpls.2016.01250

**Published:** 2016-08-30

**Authors:** Shah Fahad, Saddam Hussain, Shah Saud, Shah Hassan, Zahid Ihsan, Adnan N. Shah, Chao Wu, Muhammad Yousaf, Wajid Nasim, Hesham Alharby, Fahad Alghabari, Jianliang Huang

**Affiliations:** ^1^National Key Laboratory of Crop Genetic Improvement, MOA Key Laboratory of Crop Ecophysiology and Farming System, College of Plant Science and Technology, Huazhong Agricultural UniversityWuhan, China; ^2^College of Resources and Environment, Huazhong Agricultural UniversityWuhan, China; ^3^Department of Horticulture, Northeast Agricultural UniversityHarbin, China; ^4^Department of Agricultural Extension Education and Communication, The University of Agriculture, PeshawarPeshawar, Pakistan; ^5^Department of Arid Land Agriculture, Faculty of Meteorology, Environment and Arid Land Agriculture, King Abdul Aziz UniversityJeddah, Saudi Arabia; ^6^Department of Environmental Sciences, COMSATS Institute of Information TechnologyVehari, Pakistan; ^7^Department of Biological Sciences, Faculty of Sciences, King Abdulaziz UniversityJeddah, Saudi Arabia; ^8^Hubei Collaborative Innovation Center for Grain Industry, Yangtze UniversityHubei, China

**Keywords:** cultivars, high night temperature, morpho-physiological growth, plant growth regulators, spikelet fertility, water use efficiency

## Abstract

A 2-year experiment was conducted to ascertain the effects of exogenously applied plant growth regulators (PGR) on rice growth and yield attributes under high day (HDT) and high night temperature (HNT). Two rice cultivars (IR-64 and Huanghuazhan) were subjected to temperature treatments in controlled growth chambers and four different combinations of ascorbic acid (Vc), alpha-tocopherol (Ve), brassinosteroids (Br), methyl jasmonates (MeJA), and triazoles (Tr) were applied. High temperature severely affected rice morphology, and also reduced leaf area, above-, and below-ground biomass, photosynthesis, and water use efficiency, while increased the leaf water potential of both rice cultivars. Grain yield and its related attributes except number of panicles, were reduced under high temperature. The HDT posed more negative effects on rice physiological attributes, while HNT was more detrimental for grain formation and yield. The Huanghuazhan performed better than IR-64 under high temperature stress with better growth and higher grain yield. Exogenous application of PGRs was helpful in alleviating the adverse effects of high temperature. Among PGR combinations, the Vc+Ve+MejA+Br was the most effective treatment for both cultivars under high temperature stress. The highest grain production by Vc+Ve+MejA+Br treated plants was due to enhanced photosynthesis, spikelet fertility and grain filling, which compensated the adversities of high temperature stress. Taken together, these results will be of worth for further understanding the adaptation and survival mechanisms of rice to high temperature and will assist in developing heat-resistant rice germplasm in future.

## Introduction

The persistently rising ambient temperature is regarded as one of the most detrimental stresses among constantly changing environmental factors. It has been predicted that the global mean temperature could rise up to 2.0–4.5°C by the end of this century and the enhancement changes in minimum night temperature will more compare to maximum day temperature (IPCC, 2007). In china, climate conditions during rice growth seasons have changed dramatically, for instance, the particular area of Yangtze River Valley (YRV) is prone to high temperature especially in mid-growing season which may lead to huge yield loss, although the temperature may rarely increase more than 35°C (Matsui, 2009). Plants are sessile organisms, which cannot move to more favorable environments; consequently, plant growth and developmental processes are substantially affected, often lethally, by high temperature (HT) stress (Lobell and Field, 2007). Production of reactive oxygen species (ROS) in excesses amount is one of the major consequences of HT stress, which leads to oxidative stress (Hasanuzzaman et al., 2012, 2013). In response to HT, plants modify their metabolism in different ways particularly by producing compatible solutes, maintaining cell turgor by osmotic adjustment, and regulating the antioxidant system to re-establish the cellular redox balance and homeostasis (Valliyodan and Nguyen, 2006; Janská et al., 2010).

Although, rice possesses a comparatively higher tolerance to high temperature during vegetative stages, but on other sides its vulnerability rises to elevated temperature during the reproductive stage, particularly at flowering (Yoshida et al., 1981; Prasad et al., 2006; Jagadish et al., 2007, 2008, 2010a,b). In the past, the spatial analyses demonstrated the vulnerable periods of rice (i.e., flowering and early grain filling) overlapping with the mounting temperatures phases in countries like Bangladesh, eastern India, southern Myanmar, and northern Thailand (Maclean et al., 2002; Wassmann et al., 2009). Considering the predicted rate of increase in night temperature, the negative impact on rice production is likely to be higher in coming years with huge yield losses. High night temperatures (HNT) are generally related with increased respiration rates, reduced pollination, number of pollen germinated on the stigma, and increased spikelet sterility, which can lead to decrease in final grain yield (Mohammed and Tarpley, 2009a,b; Jagadish et al., 2010a). Likewise, high day temperatures can severely influence the photosynthesis activity, by bringing alterations in thylakoids structural organization and thus interrupting the whole cycle of photosynthetic system II (Karim et al., 1997; Zhang et al., 2005). Moreover, it results in production of excessive ROS, which affects the membrane integrity and may cause cell death (Schoffl et al., 1999; Howarth, 2005).

Various combinations of plant growth regulators (PGR) have been reported to improve the heat tolerance and stand establishment of rice (Mohammed and Tarpley, 2011; Shah et al., 2011; Fahad et al., 2015b) and no negative interaction among PGRs was observed in these studies. The ascorbic acid (vitamin C; Vc), alpha-tocopherol (vitamin E; Ve), brassinosteroids (Br), methyl jasmonates (MeJA), and triazoles (Tr) that were utilized in this experiment are pivotal in enhancing the agronomical and physiological attributes associated with thermo resistance and provide shelter against damaging influence of oxidative stress (Fletcher et al., 2000; Zhang et al., 2007; Beltagi, 2008; Mohammed and Tarpley, 2011). The Vc is a ubiquitous metabolite in plant tissues that performs a variety of functions. It plays an important role in the protection of cells and organelles from the oxidative damage by scavenging ROS, which are produced by environmental stresses such as heat. There is also growing evidence of the Vc participation in the regulation of cell division and elongation (Horemans et al., 2003). The Ve performs a vital role in the production of vital organic acids (L-tartaric, L-theronic, L-glyceric, and L-oxalic acids), and also take part in numerous processes such as flavonoid and phytohormone biosynthesis, and the xanthophyll cycle (Debolt et al., 2007). Furthermore, Ve diminishes the harmful effect of ROS and scavenges lipid peroxyl radicals (Munné-Bosch et al., 1999). Br are steroidal plant hormones, which perform a key role in various cellular and physiological processes like stem elongation, pollen tube growth, root inhibition, fruit development, ethylene biosynthesis, proton pump activity, xylem differentiation, photosynthesis, and gene expression (Yu et al., 2004; Fu et al., 2008). The Br can stimulate plant tolerance to a variety of abiotic stresses, such as high and low temperatures, drought, and salinity injury (Krishna, 2003; Kagale et al., 2007). It has been found that Br-induced increase in the basic thermo-tolerance was associated with increased heat shock protein synthesis and accumulation as well as increased expression of some components of translational machinery (Dhaubhadel et al., 2002).

The MeJA are crucial cellular regulators that are involved in several plant developmental processes in plants (Ueda and Saniewski, 2006; Norastehnia et al., 2007). Foliar application of MeJA brings alteration in numerous physiological responses and thus stimulates plant defense responses against a variety of biotic and abiotic stresses (Walia et al., 2007). Clarke et al. (2009) reported that exogenous application of MeJA in *A. thaliana* conferred basal thermo-tolerance and protected from the damaging influence of heat shock. Triazoles (Tr), as a plant growth regulating properties have the capability to cause changes in the balance of important plant hormones against stress including cytokinin augmentation, increased ABA and reduced ethylene (Kamountsis and Chronopoulon-Sereli, 1999; Fletcher et al., 2000; Gopi et al., 2007; Hajihashemi et al., 2007). Fletcher et al. (2000) observed that Tr-treated bean plants recorded less electrolyte leakage and were tolerant against heat stress.

Comparative responses of rice to either high-day time or night-time temperature stress in terms of physiological processes and reproductive function are not well understood. Furthermore, the different combinations of PGR (Ve, Vc, Br, MeJA, Tr) used in the present study have never been tested against high temperature stress in the past. Therefore, the present study was carried out to ascertain the morpho-physiological and yield responses of two rice cultivars to exogenously applied PGRs under high day and HNT stress. The findings of the present study will strengthen our understanding to improve the adaptation, survival and tolerance of rice to high temperature.

## Materials and methods

### Plant husbandry and growth conditions

Present studies were carried out in a greenhouse at the Huazhong Agricultural University, Wuhan, China (30° 47′N, 114° 35′E), during 2013 and 2014 growing seasons. IR-64 and Huanghuazhan (HHZ) cultivars were used in the present study. Both belong to indica rice (*Oryza sativa* L.) cultivar and have approximately akin plant architecture (medium stature) however contradictory responses to temperature. The HHZ is tolerant to high temperature stress, while IR-64 sensitive to high temperature (Fahad et al., 2015a,b, 2016). Rice plants were normally grown under natural conditions till booting stage. To facilitate germination, a wet towel was used to maintain the seeds' moisture for 2 days. Different seedling trays were used after germination, and the seeds were subsequently placed in each cell (1 seed per cell). Three seedlings were transplanted after sowing of 3 weeks were transplanted to plastic pots (21.6 cm lower inside diameter, 27.2 cm upper inner diameter, 27.2 cm height and 0.15 cm thickness) filled with 12 kg of air-dried soil. In both years, IR-64 was sown for 12 more days than HHZ to match the heading dates of these varieties. The soil was silt loam containing sand, silt and clay at 32, 54, and 14%, respectively. To each pot, 10 g of compound fertilizer (16% N: 16% P: 16% K) was applied. Standard guidelines for pot experiments were followed and no pest or disease problem was found during the experimental period.

### Treatments

For temperature treatments, three indoor controlled environment growth chambers (Climatrons, Southeast Ningbo Instruments Ltd, Zhejiang, China) already set at three different temperatures treatment, i.e., HDT (high day temperature of 35°C ± 2; night temperature: 28 ± 2°C), HNT (HNT of 32°C ± 2; day temperature: 28 ± 2°C) and AT (ambient temperature of 28°C ± 2 throughout the day) were provided. HDT initiated at 7 a.m. to 7 p.m., while HNT started at 7 p.m. and lasted for 12 h daily (7 p.m. to 7 a.m.). Control (AT) plants were grown at 28°C (12 h-day/12 h-night cycles). The heat treatments were started from booting stage (as most of the damage to rice caused by high temperature occurs between these durations) to physiological maturity of plants. During whole duration, humidity level was maintained at level of 75% constantly while photosynthetic photon flux density kept at 1000 μM m^−2^ s^−1^ within the growth chamber. However, CO_2_ was not determined inside the chamber.

Different combinations of PGR were exogenously applied three times at 30, 35, and 40 days after emergence (DAE) to enable thorough coverage earlier to imposing heat stress. Various PGR treatments that used throughout the experiment were, [1] vitamin C + vitamin E + methyl jasmonates + brassinosteroids (Vc+Ve+MeJA+Br), [2] brassinosteroids + triazoles + methyl jasmonates (Br+Tr+MeJA), [3] vitamin C + vitamin E (Vc+Ve), [4] methyl jasmonates (MeJA), and [5] nothing applied control (NAC). The Vc, Ve, MeJA, Br and Tr were applied at the rate of 1.4, 6.9, 1.8, 4.0, and 0.55 ppm solution, accordingly in respective treatments. De-ionized water was used in order to dissolve Vc, nevertheless; minute amount of ethyl alcohol was firstly used to dissolve Ve and after that de-ionized water was further added to make the volume up to desirable mark. Both Vc and Vc were purchased from Sigma-Aldrich, Shanghai, China and Br, MeJA, and Tr were supplied by Olchemim Ltd, Czech Republic.

### Observations

#### Gas exchange and plant water relations

The photosynthesis (*A*), stomatal conductance (*gs*), internal CO_2_ concentration (*Ci*), and transpiration (*E*) were measured in both years between 1000 and 1200 h, on the penultimate leaves using a LI-6400 portable photosynthesis system (LI-COR Inc., Lincoln, NE, USA) during heading stage of both rice varieties. Water use efficiency (WUE) and stomatal limitations to CO_2_ uptake were also observed. In this study, WUE is a ratio between *A* and E while stomatal limitations are the ratio between A and Ci (Farooq et al., 2009).

To measure water potential of leaf, flag leaf was detached from each treated rice plants during heading stage. After that it was cut into tiny slices, and straight away followed by leaf potential measurement using a WP4C Dew point potential meter (Decagon Devices Inc., Pullman, WA, USA) (Fahad et al., 2016).

#### Morphology and dry weights

At panicle and heading stages, dry weights of above-ground total biomass and roots were determined. Viable leaf area per plant at all the three stages and total green leaf area at maturity time were determined using a LI-3100C area meter (LI-COR Inc., Lincoln, NE, USA). Plant height, leaf length (start measuring from the position of leaf blade contact and the stem to the tip of the leaf) and width (widest part of the leaf) of the penultimate leaf was also measured.

#### Yield and its components

In order to measure grain yield and yield components at the time of maturity during both year experiments, all the hills were collected from each treated pot. Number of panicle was counted from each collected plant to calculate its total number panicle per pot. Plants were then separated into straw and panicles. Straw was further divided into leaves and stems which were then oven dried at 80°C to constant weight and then dry weights were calculated. Panicle length at maturity stage was determined. Individual panicle weight from main tiller was measured. The panicles were hand-threshed and filled spikelets were separated from unfilled spikelets through a seed blower. Grains were soaked in tap water and the numbers of sunken and floating grains were counted and weighed to determine three yield components: the number of spikelets per panicle, the filled grain percentage and the 1000-grain weight (Fahad et al., 2016). Primary and secondary branches were also calculated. According to Mohammed and Tarpley (2011) spikelet fertility was determined. During our experiment spikelet fertility was described as the ratio of filled grains number to the entire amount of reproductive sites (florets) during ANT treatment. This technique was used because during HDT and HNT treatments most of the abortions of florets were observed. In order to find out whether the grain was filled or empty, every floret was force downed between the thumb and forefinger. Filled grains number comprised both fully and half-filled grains. Spikelet fertility was expressed as percentage.

#### Statistical analysis

For each rice cultivar and year, the experiment was arranged in a completely randomized design with two factors viz., temperature treatments and PGRs. Each treatment was replicated four times in each year. Data collected was analyzed with the analysis of variance technique (ANOVA) and presented as the interaction of temperature treatments and PGRs. Statistical analysis was performed by software Statistix 9.0 (Analytical Software, Tallahassee, FL, USA) using HSD tukey's test (*P* ≤ 0.05).

## Results

### Thermoregulation of leaf physiology

Rice leaf physiological attributes were negatively affected under high day and night temperature treatments applied at booting stage of crop, but their interactions were non-significant (*p* > 0.05) for most of the time in both years (Tables 1, 2). Significant (*p* ≤ 0.05) variations for *A, gs*, A/Ci, WUE and LWP were observed in two consecutive years under the influence of high temperature and PGRs application in both rice cultivars. The E and Ci were also significantly affected by main effects of both factors during first year (Table 1). Interaction of temperature × PGR was non-significant (*p* > 0.05) for all physiological attributes except E for both years (Tables 1, 2).

**Table 1:** **The effects of high temperature stress and exogenously applied plant growth regulators on the photosynthesis (*A*), stomatal conductance (*gs*), transpiration (*E*), intercellular CO_2_ concentration (*Ci*), stomatal limitations to CO_2_ uptake (A/Ci), water use efficiency (A/E), and leaf water potential in two rice cultivars during 2013 growing season**.

**Temp**.	**PGR**	***A* (mmol m^−2^ s^−1^)**		***gs* (mol m^−2^ s^−1^)**		***E* (mmol m^−2^ s−1)**		***Ci* (mmol mol^−1^)**		**Stomatal limitations to CO_2_ uptake (A/Ci)**		**Water use efficiency (A/E)**		**Water potential of leaves-Mpa**	
		**IR-64**	**HHZ**	**IR-64**	**HHZ**	**IR-64**	**HHZ**	**IR-64**	**HHZ**	**IR-64**	**HHZ**	**IR-64**	**HHZ**	**IR-64**	**HHZ**
HNT	Vc+Ve+MeJA+Br	18.13	22.58	0.75	0.97	10.47	12.31	289.7	315.2	0.063	0.072	1.74	1.84	1.15	1.19
	Br+Tr+MeJA	16.68	21.01	0.85	1.03	11.25	12.64	298.2	322.6	0.056	0.065	1.49	1.66	1.23	1.26
	Vc+Ve	17.97	22.24	0.78	1.00	10.83	12.42	291.5	318.7	0.062	0.070	1.66	1.80	1.19	1.22
	MeJA	16.06	20.63	0.91	1.08	12.54	13.06	303.5	328.0	0.053	0.063	1.28	1.58	1.28	1.29
	NAC	15.55	19.72	0.94	1.09	12.69	13.16	306.6	330.7	0.051	0.060	1.23	1.50	1.31	1.32
HDT	Vc+Ve+MeJA+Br	17.47	22.34	1.05	1.11	11.37	12.77	303.3	325.2	0.058	0.069	1.54	1.75	1.29	1.30
	Br+Tr+MeJA	15.60	19.60	1.14	1.19	11.98	13.10	309.2	329.4	0.051	0.060	1.30	1.50	1.39	1.34
	Vc+Ve	17.27	21.75	1.10	1.14	11.83	12.85	306.1	326.8	0.057	0.067	1.46	1.69	1.34	1.31
	MeJA	13.76	19.31	1.19	1.22	12.83	13.74	311.9	334.8	0.044	0.058	1.07	1.41	1.49	1.38
	NAC	13.13	18.93	1.25	1.25	13.14	13.92	316.2	337.0	0.042	0.056	1.00	1.36	1.53	1.41
AT	Vc+Ve+MeJA+Br	22.58	23.12	0.62	0.87	9.37	12.28	272.9	312.7	0.083	0.074	2.41	1.89	0.79	0.90
	Br+Tr+MeJA	20.14	22.08	0.69	0.92	9.68	12.48	282.6	316.8	0.071	0.070	2.08	1.77	0.84	0.95
	Vc+Ve	22.18	23.01	0.65	0.89	9.56	12.34	276.7	315.3	0.080	0.073	2.32	1.87	0.81	0.92
	MeJA	18.97	21.52	0.73	0.97	10.10	12.60	288.7	318.3	0.066	0.068	1.88	1.71	0.87	0.99
	NAC	18.35	20.94	0.76	1.00	10.25	12.92	292.5	320.0	0.063	0.066	1.79	1.62	0.89	1.02
Temperature		^**^	^**^	^**^	^**^	^**^	^**^	^**^	^**^	^**^	^**^	^**^	^**^	^**^	^**^
PGR		^*^	^**^	^**^	^**^	^**^	^*^	^*^	^*^	^**^	^*^	^**^	^**^	^**^	^*^
Temperature × PGR		ns	ns	ns	ns	^*^	^*^	ns	ns	ns	ns	ns	ns	ns	ns
CV		6.78	6.93	10.45	6.97	6.16	6.52	6.14	6.51	6.88	6.29	7.44	7.45	4.46	5.51

^**^ and ^*^ denote significance at the 0.01 and 0.05 probability level, respectively. ns, non-significant; CV, Coefficient of variation; PGR, Plant growth regulators; HHZ, Huanghuazhan (heat tolerant), IR-64 (heat susceptible). HDT, high day temperature; HNT, high night temperature; AT, ambient temperature (control). Different PGRs viz., vitamin C (Vc), vitamin E (Ve), methyl jasmonates (MeJA), brassinosteroids (Br) and triazoles (Tr) were applied at the rate of 1.4, 6.9, 1.8, 4.0, and 0.55 ppm solution, respectively in respective treatments; NAC, nothing applied control.

**Table 2:** **The effects of high temperature stress and exogenously applied plant growth regulators on the photosynthesis (*A*), stomatal conductance (*gs*), transpiration (*E*), intercellular CO_2_ concentration (*Ci*), stomatal limitations to CO_2_ uptake (A/Ci), water use efficiency (A/E), and leaf water potential in two rice cultivars during 2014 growing season**.

**Temp**.	**PGR**	***A* (mmol m^−2^ s^−1^)**		***gs* (mol m^−2^ s^−1^)**		***E* (mmol m^−2^ s^−1^)**		***Ci* (mmol mol^−1^)**		**Stomatal limitations to CO_2_ uptake (A/Ci)**		**Water use efficiency (A/E)**		**Water potential of leaves -Mpa**	
		**IR-64**	**HHZ**	**IR-64**	**HHZ**	**IR-64**	**HHZ**	**IR-64**	**HHZ**	**IR-64**	**HHZ**	**IR-64**	**HHZ**	**IR-64**	**HHZ**
HNT	Vc+Ve+MeJA+Br	17.64	21.41	0.63	0.86	10.33	12.15	286.4	311.1	0.062	0.069	1.71	1.77	1.30	1.33
	Br+Tr+MeJA	16.32	19.84	0.72	0.91	11.10	12.49	294.5	318.2	0.055	0.062	1.48	1.59	1.36	1.41
	Vc+Ve	17.47	21.09	0.65	0.89	10.69	12.26	288.2	314.6	0.061	0.067	1.64	1.72	1.31	1.37
	MeJA	15.65	19.48	0.79	0.96	12.39	12.91	300.1	323.9	0.052	0.060	1.27	1.51	1.41	1.42
	NAC	15.22	18.53	0.81	0.98	12.55	13.01	303.3	326.4	0.050	0.057	1.22	1.43	1.46	1.47
HDT	Vc+Ve+MeJA+Br	17.14	21.19	0.93	1.00	11.23	12.62	300	320.6	0.057	0.066	1.53	1.68	1.44	1.45
	Br+Tr+MeJA	14.95	18.45	1.01	1.07	11.84	12.94	305.8	325.1	0.049	0.057	1.27	1.43	1.53	1.49
	Vc+Ve	16.53	20.6	0.97	1.03	11.69	12.70	302.8	322.5	0.055	0.064	1.41	1.62	1.48	1.46
	MeJA	13.57	18.16	1.07	1.10	12.69	13.59	308.5	330.5	0.044	0.055	1.07	1.34	1.62	1.54
	NAC	13.23	17.72	1.12	1.14	12.99	13.76	312.8	332.6	0.042	0.053	1.02	1.29	1.67	1.56
AT	Vc+Ve+MeJA+Br	22.23	22.62	0.50	0.76	9.22	12.13	269.5	308.4	0.082	0.073	2.41	1.87	0.93	1.04
	Br+Tr+MeJA	19.85	21.58	0.56	0.81	9.54	12.32	279.3	312.4	0.071	0.069	2.08	1.75	0.84	0.95
	Vc+Ve	21.72	22.51	0.53	0.78	9.42	12.18	273.4	311	0.079	0.072	2.31	1.85	0.81	0.94
	MeJA	18.51	21.02	0.60	0.86	9.96	12.44	285.4	314	0.065	0.067	1.86	1.69	0.87	0.99
	NAC	18.12	20.41	0.63	0.88	10.11	12.76	289.1	315.7	0.063	0.065	1.79	1.60	0.89	1.02
Temperature		^**^	^**^	^**^	^**^	^**^	^**^	^**^	^**^	^**^	^**^	^**^	^**^	^**^	^**^
PGR		^**^	^**^	^**^	^**^	^**^	ns	^*^	ns	^**^	^*^	^*^	^**^	^*^	^*^
Temperature × PGR		ns	ns	ns	ns	^*^	^*^	ns	ns	ns	ns	ns	ns	ns	ns
CV		5.67	5.98	10.45	6.97	5.62	5.45	6.14	7.49	5.22	5.68	9.98	7.45	5.27	7.13

^**^ and ^*^ denote significance at the 0.01 and 0.05 probability level, respectively. ns, non-significant; CV, Coefficient of variation; PGR, Plant growth regulators; HHZ, Huanghuazhan (heat tolerant), IR-64 (heat susceptible). HDT, high day temperature; HNT, high night temperature; AT, ambient temperature (control). Different PGRs viz., vitamin C (Vc), vitamin E (Ve), methyl jasmonates (MeJA), brassinosteroids (Br) and triazoles (Tr) were applied at the rate of 1.4, 6.9, 1.8, 4.0, and 0.55 ppm solution, respectively in respective treatments; NAC, nothing applied control.

High temperature stress hampered rice photosynthesis machinery through limiting stomatal CO_2_ uptake and enhancing gs, E and Ci. Averaged across years, cultivars and PGR combinations, a reduction of 17.11 and 14.02% in *A* and 22.09 and 14.66% in *A/Ci* was recorded in rice plants under HDT and HNT stress, respectively (Tables 1, 2). Both cultivars behaved variably to high temperature stress and furnished significant level of tolerance to HDT and HNT. The cultivar HHZ performed better all physiological traits than IR-64 except gs which was similar in both cultivars. Under high temperature, *A* and *A/Ci* were decreased by 21.21 and 26.42% in IR-64, and 9.92 and 10.34% in HHZ, respectively compared with AT (Tables 1, 2). Rice plants under HDT recorded highest gs (1.25 mol m^−2^ s^−1^), E (13.92 mmol m^−2^ s^−1^) and Ci (337 mmol mol^−1^). Inverse relationship in WUE and LWP under high temperature stress was measured. Overall, reduction of 28.89 and 20.15% in WUE and improvement of 62.22 and 49.14% in LWP were noted under HDT and HNT, respectively (Tables 1, 2).

All PGR combinations effectively enhanced the stress tolerance in both cultivars (Tables 1, 2). The PGR combination of Vc+Ve improved plant physiological attributes up to 46% under HDT and 34.96% under HNT and addition of MeJA and Br to Vc+Ve combination (Vc+Ve+MeJA+Br) further improved stress tolerance to 54% under HDT and 41.43% under HNT. Different combinations of PGRs improved *A* by 10.62, 15.83, and 10.13% under AT, HDT, and HNT, respectively compared with NAC (Tables 1, 2). The PGRs application increased WUE and *A/Ci* by 40.71 and 35.71% compared with NAC. The Vc+Ve+MeJA+Br outcompeted all other combinations of PGR for all physiological traits particularly under HDT and HNT stress (Tables 1, 2).

### Anatomy and growth attributes

Rice leaf anatomy and growth attributes responded variably to HDT and HNT stress cycles. Leaf length, panicle length, above- (AGB) and below-ground biomass (RB), and total green leaf area (TGLA) were significantly (*p* ≤ 0.05) affected by various temperature treatments, however, plant height and leaf width were unaffected (Tables 3, 4). The PGR combinations also significantly (*p* ≤ 0.05) influenced all the growth attributes except plant height and leaf width. Interaction of temperature × PGR was non-significant (*p* > 0.05) for all above mentioned leaf and growth parameters. Compared with AT, the HDT and HNT reduced the panicle length of IR-64 by 28.27 and 15.49%, leaf length of IR-64 by 15.44 and 7.46%, the panicle length of HHZ by 11.66 and 4.26% and leaf length of HHZ by 9.79 and 4.78%, respectively averaged across years and PGRs (Tables 3, 4). The AGB, RB, and TGLA at maturity also presented similar trend as for other crop growth attributes. The AT resulted in maximum AGB, RB and TGLA, while HNT reduced AGB by 15.96 and 8.47%, RB by 27.8 and 7.5% and TGLA by 24.87 and 12.47% in IR-64 and HHZ, respectively (Tables 3, 4). The corresponding reductions by HDT were almost double to HNT; and AGB was decreased by 33.55 and 18.76%, RB by 42.36 and 24.19%, and TGLA by 32.35 and 19.82% in IR-64 and HHZ, respectively (Tables 3, 4). The year effect was non-significant (*p* > 0.05) for all these attributes.

**Table 3:** **The effects of high temperature stress and exogenously applied plant growth regulators on different growth attributes of two rice cultivars during 2013 growing season**.

**Temp**.	**PGR**	**Plant height (cm)**		**Panicle length (cm)**		**Leaf length (cm)**		**Leaf width (cm)**		**AGB (g pot^−1^)**		**RB (g pot^−1^)**		**TGLA (cm^2^)**	
		**IR-64**	**HHZ**	**IR-64**	**HHZ**	**IR-64**	**HHZ**	**IR-64**	**HHZ**	**IR-64**	**HHZ**	**IR-64**	**HHZ**	**IR-64**	**HHZ**
HNT	Vc+Ve+MeJA+Br	116.8	120.3	24.0	27.3	42.0	47.5	1.3	1.4	42.3	52.6	8.43	10.80	168.5	240.1
	Br+Tr+MeJA	117.3	121.8	21.8	26.0	39.5	44.8	1.3	1.4	38.3	49.5	8.18	10.35	152.2	226.7
	Vc+Ve	116.8	120.8	22.8	26.5	40.8	46.0	1.4	1.4	40.9	51.5	8.41	10.57	162.4	230.2
	MeJA	119.3	122.0	20.3	25.5	37.8	43.3	1.3	1.3	36.2	47.0	7.96	10.12	139.4	216.6
	NAC	119.8	123.3	19.3	24.0	36.3	42.0	1.3	1.4	34.8	46.8	7.87	9.90	129.6	198.7
HDT	Vc+Ve+MeJA+Br	116.3	119.5	21.8	25.5	38.5	46.3	1.3	1.4	33.5	47.0	7.48	9.01	158.2	226.8
	Br+Tr+MeJA	117.8	120.8	19.5	23.8	35.3	43.0	1.3	1.4	28.7	42.5	6.88	8.67	138.3	209.8
	Vc+Ve	116.8	120.5	20.5	24.3	37.0	44.5	1.3	1.4	31.8	44.9	7.21	8.90	149.8	219.2
	MeJA	118.5	121.8	18.0	22.8	32.5	41.0	1.2	1.3	26.0	40.9	6.20	8.44	128.9	196.9
	NAC	118.8	123.0	16.0	22.0	33.0	39.8	1.2	1.3	25.5	41.4	6.09	8.35	116.7	182.0
AT	Vc+Ve+MeJA+Br	113.0	118.8	25.8	27.5	42.5	47.8	1.4	1.4	47.8	55.6	13.03	12.26	213.1	256.3
	Br+Tr+MeJA	114.0	120.0	24.0	26.3	40.3	45.8	1.4	1.4	47.1	53.9	12.55	12.12	190.4	239.8
	Vc+Ve	114.0	120.0	25.0	26.8	41.5	46.8	1.4	1.4	47.7	55.3	12.84	12.23	201.5	251.0
	MeJA	114.3	120.5	23.8	25.5	39.8	44.8	1.4	1.4	45.5	53.3	12.33	11.99	180.4	234.1
	NAC	115.0	121.3	22.8	25.0	39.0	44.0	1.4	1.4	44.2	52.2	12.04	11.87	172.5	227.0
Temperature		ns	ns	^*^	^*^	^**^	^**^	^*^	ns	^**^	^**^	^*^	^**^	^**^	^**^
PGR		ns	ns	^*^	ns	^**^	^*^	ns	ns	^*^	^*^	^*^	^*^	^**^	^**^
Temperature × PGR		ns	ns	ns	ns	ns	ns	ns	ns	ns	ns	ns	ns	ns	ns
CV		7.23	8.54	7.96	7.49	6.59	7.41	4.38	5.03	4.28	5.67	6.82	5.09	6.48	7.23

^**^ and ^*^ denote significance at the 0.01 and 0.05 probability level, respectively. ns, non-significant; CV, Coefficient of variation; PGR, Plant growth regulators; HHZ, Huanghuazhan (heat tolerant), IR-64 (heat susceptible). HDT, high day temperature; HNT, high night temperature; AT, ambient temperature (control). AGB, aboveground biomass; RB, root biomass; TGLA, total green leaf area at maturity. Different PGRs viz., vitamin C (Vc), vitamin E (Ve), methyl jasmonates (MeJA), brassinosteroids (Br) and triazoles (Tr) were applied at the rate of 1.4, 6.9, 1.8, 4.0, and 0.55 ppm solution, respectively in respective treatments; NAC, nothing applied control. Above ground and root dry biomass was recorded at panicle initiation stage.

**Table 4:** **The effects of high temperature stress and exogenously applied plant growth regulators on different growth attributes of two rice cultivars during 2014 growing season**.

**Temp**.	**PGR**	**Plant height (cm)**		**Panicle length (cm)**		**Leaf length (cm)**		**Leaf width (cm)**		**AGB (g pot^−1^)**		**RB (g pot^−1^)**		**TGLA (cm^2^)**	
		**IR-64**	**HHZ**	**IR-64**	**HHZ**	**IR-64**	**HHZ**	**IR-64**	**HHZ**	**IR-64**	**HHZ**	**IR-64**	**HHZ**	**IR-64**	**HHZ**
HNT	Vc+Ve+MeJA+Br	116.0	118.0	25.3	28.5	42.5	47.8	1.4	1.4	43.0	53.1	11.99	15.59	179.3	250.8
	Br+Tr+MeJA	117.8	120.0	23.0	27.3	40.0	45.3	1.4	1.4	41.0	51.8	11.14	14.91	163.0	237.4
	Vc+Ve	116.0	118.5	24.0	27.8	41.3	46.5	1.4	1.4	42.1	52.6	11.76	15.37	173.3	240.9
	MeJA	119.5	122.3	21.5	26.8	38.3	43.8	1.4	1.4	40.5	51.5	11.00	14.63	150.3	227.2
	NAC	120.5	123.8	20.5	25.3	36.8	42.5	1.4	1.4	40.3	50.8	10.81	14.46	140.4	209.3
HDT	Vc+Ve+MeJA+Br	114.3	119.0	23.5	27.0	39.3	46.8	1.3	1.4	32.8	49.1	9.65	12.66	169.1	237.4
	Br+Tr+MeJA	116.8	120.0	21.3	25.3	36.0	43.5	1.3	1.4	31.1	46.9	8.92	11.75	149.2	220.4
	Vc+Ve	115.8	119.0	22.3	25.8	37.8	45.0	1.3	1.4	32.0	48.6	9.40	12.27	160.7	229.9
	MeJA	118.0	120.8	19.8	24.3	33.3	41.5	1.3	1.3	30.7	45.9	8.49	11.45	139.7	207.6
	NAC	119.8	122.8	17.8	23.5	33.8	40.3	1.2	1.3	30.1	45.2	8.33	11.15	127.5	192.6
AT	Vc+Ve+MeJA+Br	117.0	117.5	27.3	29.0	43.3	48.5	1.4	1.5	49.1	56.7	14.60	15.01	221.8	264.9
	Br+Tr+MeJA	116.5	119.0	25.5	27.8	41.0	46.5	1.4	1.4	48.6	56.0	14.32	14.80	199.1	248.4
	Vc+Ve	117.0	118.0	26.5	28.3	42.3	47.5	1.4	1.5	48.9	56.4	14.47	14.92	210.1	259.6
	MeJA	116.0	119.8	25.3	27.0	40.5	45.5	1.4	1.4	48.5	55.8	14.24	14.67	189.1	242.7
	NAC	114.8	121.0	24.3	26.5	40.0	44.8	1.4	1.4	48.0	55.7	13.93	14.85	181.2	235.6
Temperature		ns	ns	^**^	^**^	^**^	^**^	ns	ns	^**^	^*^	^**^	^**^	^**^	^**^
PGR		ns	^*^	^*^	^*^	^*^	^*^	ns	ns	^*^	^*^	^*^	^*^	^*^	^**^
Temperature × PGR		ns	ns	ns	ns	ns	ns	ns	ns	ns	ns	ns	ns	ns	ns
CV		8.34	6.53	5.43	5.38	5.59	6.11	10.18	9.32	4.32	6.41	5.21	5.37	9.13	6.89

^**^ and ^*^ denote significance at the 0.01 and 0.05 probability level, respectively. ns, non-significant; CV, Coefficient of variation; PGR, Plant growth regulators; HHZ, Huanghuazhan (heat tolerant), IR-64 (heat susceptible). HDT, high day temperature; HNT, high night temperature; AT, ambient temperature (control). AGB, aboveground biomass; RB, root biomass; TGLA, total green leaf area at maturity. Different PGRs viz., vitamin C (Vc), vitamin E (Ve), methyl jasmonates (MeJA), brassinosteroids (Br) and triazoles (Tr) were applied at the rate of 1.4, 6.9, 1.8, 4.0, and 0.55 ppm solution, respectively in respective treatments, NAC, nothing applied control. Above ground and root dry biomass was recorded at panicle initiation stage.

Various PGRs combinations alleviated the heat-induced effects by improving panicle length, leaf length, AGB, RB and TGLA (Tables 3, 4). The plant height and leaf width were unaffected by PGRs. This improvement was greater in IR-64 for ABG and TGLA while lower for RB over HHZ. The PGRs were more effective under HDT; higher increase in ABG, RB and TGLA was observed by different PGR combination under HDT as compared with NAC (Tables 3, 4). The Vc+Ve+MeJA+Br was the most effective combination than all other treatments. This combination increased TGLA by 25, 26, and 18% under HNT, HDT and AT, respectively irrespective of the cultivar. The Ve+Vc was second best treatment for these attributes (Tables 3, 4).

### Rice yield and yield components

Rice grain yield and yield contributors viz., number of panicle per plant, spikelet per panicle, secondary branches per panicle, spikelet fertility, grain filling percentage and 1000-grain weight were significantly (*p* ≤ 0.05) affected by high temperature stress (Tables 5, 6). The number of primary branches per panicle was unaffected by temperature treatments. Effect of PGR combinations was also significant (*p* = 0.05) for above mentioned attributes except number of panicles per plant and primary branches per panicle in both years (Tables 5, 6). Interaction of temperature × PGR was non-significant (*p* > 0.05) in both genotypes for successive years. An increment in number of panicle per plant was recorded under HDT and HNT and such an improvement was higher in IR-64. All other grain yield contributors presented remarkable reduction under high temperature stress particularly under HNT (Tables 5, 6). The HDT and HNT reduced 1000-grain weight by 2.95 and 5.45% and grain filling percentage by 11.43 and 47.22%. The HNT recorded almost 50% higher yield loss compared with HDT. The IR-64 was more sensitive to high temperature than HHZ and recorded 72.2 and 43.2% less grain yield under HNT and HDT, while grain yield of HHZ was reduced by 36.44 and 25.36%, respectively (Tables 5, 6).

**Table 5:** **The effects of high temperature stress and exogenously applied plant growth regulators on grain yield and its related components in two rice cultivars during 2013 growing season**.

**Temp**.	**PGR**	**Panicles (plant^−1^)**		**Spikelet (Panicle^−1^)**		**Primary branches (# panicle^−1^)**		**Secondary branches (# panicle^−1^)**		**Spikelet fertility (%)**		**Grain filling (%)**		**1000-GW (g)**		**Grain yield (g plant^−1^)**	
		**IR-64**	**HHZ**	**IR-64**	**HHZ**	**IR-64**	**HHZ**	**IR-64**	**HHZ**	**IR-64**	**HHZ**	**IR-64**	**HHZ**	**IR-64**	**HHZ**	**IR-64**	**HHZ**
HNT	Vc+Ve+MeJA+Br	41.3	38.5	99.0	135.8	12.8	13.3	42.3	45.5	47.3	66.2	40.2	69.1	19.0	20.8	22.6	45.0
	Br+Tr+MeJA	43.0	39.8	94.8	130.8	12.8	12.8	37.5	41.3	43.4	62.7	36.6	66.3	18.8	20.5	19.5	41.9
	Vc+Ve	42.0	39.3	96.3	133.0	13.0	13.3	39.8	43.0	44.9	64.2	38.5	67.1	18.8	20.8	20.9	43.3
	MeJA	43.3	40.5	91.8	129.5	12.8	13.3	36.0	39.8	41.8	61.7	34.6	64.0	18.5	20.3	15.0	39.7
	NAC	44.0	42.0	89.0	128.0	12.8	12.8	35.0	39.0	39.9	60.5	33.9	63.8	18.5	20.3	13.9	39.6
HDT	Vc+Ve+MeJA+Br	39.0	37.5	109.0	143.3	12.8	13.3	44.8	47.5	62.7	73.4	43.2	67.8	19.8	21.3	36.2	53.7
	Br+Tr+MeJA	41.5	38.0	102.8	138.5	12.8	12.8	41.3	43.8	59.3	70.3	40.3	68.8	19.5	21.0	32.4	49.0
	Vc+Ve	40.3	37.5	105.5	141.8	13.0	13.3	42.8	45.3	60.6	71.7	41.0	70.3	19.8	21.0	34.4	51.2
	MeJA	40.3	38.3	98.3	136.3	13.0	13.3	39.0	42.0	57.8	66.4	39.1	67.0	19.3	20.8	30.2	47.1
	NAC	42.7	39.0	95.5	133.8	12.8	12.8	37.8	39.8	54.4	67.8	38.2	66.8	19.0	20.5	28.4	46.5
AT	Vc+Ve+MeJA+Br	32.0	35.0	126.5	151.8	12.8	13.3	50.5	51.5	79.4	90.0	68.6	84.2	20.5	21.3	53.0	67.2
	Br+Tr+MeJA	33.3	35.5	124.0	148.3	12.8	12.8	47.3	48.5	77.4	88.1	65.8	82.0	20.3	21.0	51.4	64.5
	Vc+Ve	32.0	35.0	125.0	150.0	12.8	13.3	49.0	50.5	78.8	88.7	67.6	83.0	20.5	21.3	52.5	65.8
	MeJA	34.8	36.5	120.3	146.0	13.0	13.3	45.3	47.3	76.8	87.5	64.7	81.3	20.0	21.0	50.7	63.0
	NAC	35.8	38.0	117.0	145.3	12.8	12.8	44.3	46.5	75.6	86.4	64.1	80.5	20.0	21.0	50.0	62.3
Temperature		^**^	^**^	^**^	^**^	ns	ns	^**^	^**^	^**^	^**^	^**^	^**^	^*^	^*^	^**^	^**^
PGR		ns	ns	^**^	ns	ns	^*^	^**^	^*^	^**^	ns	^*^	ns	^*^	^*^	^*^	^**^
Temperature × PGR		ns	ns	ns	ns	ns	ns	ns	ns	ns	ns	ns	ns	ns	ns	ns	ns
CV		10.1	9.7	5.7	6.2	5.1	4.7	5.3	5.6	6.4	7.2	6.9	5.7	6.6	5.9	10.2	7.8

^**^ and ^*^ denote significance at the 0.01 and 0.05 probability level, respectively. ns, non-significant; CV, Coefficient of variation; PGR, Plant growth regulators; HHZ, Huanghuazhan (heat tolerant), IR-64 (heat susceptible). HDT, high day temperature; HNT, high night temperature; AT, ambient temperature (control). Different PGRs viz., vitamin C (Vc), vitamin E (Ve), methyl jasmonates (MeJA), brassinosteroids (Br) and triazoles (Tr) were applied at the rate of 1.4, 6.9, 1.8, 4.0, and 0.55 ppm solution, respectively in respective treatments, NAC, nothing applied control.

**Table 6:** **The effects of high temperature stress and exogenously applied plant growth regulators on grain yield and its related components in two rice cultivars during 2014 growing season**.

**Temp**.	**PGR**	**Panicles (plant^−1^)**		**Spikelet (Panicle^−1^)**		**Primary branches of panicle (#)**		**Secondary branches of panicle (#)**		**Spikelet fertility (%)**		**Grain filling (%)**		**1000-GW (g)**		**Grain yield (g plant^−1^)**	
		**IR-64**	**HHZ**	**IR-64**	**HHZ**	**IR-64**	**HHZ**	**IR-64**	**HHZ**	**IR-64**	**HHZ**	**IR-64**	**HHZ**	**IR-64**	**HHZ**	**IR-64**	**HHZ**
HNT	Vc+Ve+MeJA+Br	38.0	37.4	104.3	140.5	13.5	13.8	43.3	46.8	55.3	72.4	45.3	74.1	19.5	22.0	29.1	50.4
	Br+Tr+MeJA	40.5	38.7	100.0	135.5	13.5	13.3	38.5	42.5	51.5	68.9	40.1	71.3	19.0	21.5	26.2	47.3
	Vc+Ve	39.2	38.5	101.5	137.8	13.5	13.8	40.8	44.3	52.9	70.5	45.6	72.1	19.3	21.8	27.4	48.7
	MeJA	40.5	39.5	97.0	134.3	13.3	13.8	37.0	41.0	49.8	67.9	40.2	69.2	18.8	21.5	21.5	45.1
	NAC	40.8	41.0	94.3	132.8	13.5	13.3	36.0	40.3	47.9	66.7	39.4	68.4	18.8	21.3	20.6	45.0
HDT	Vc+Ve+MeJA+Br	36.3	37.3	114.3	148.0	13.5	13.8	47.0	48.8	70.8	79.7	48.3	72.3	20.3	22.5	43.0	59.1
	Br+Tr+MeJA	38.0	37.8	108.0	143.3	13.8	13.3	42.3	45.0	67.4	76.5	45.4	73.9	19.5	22.3	39.1	54.3
	Vc+Ve	37.3	37.3	110.8	146.5	13.8	13.8	44.0	46.8	68.6	77.9	46.3	75.3	20.0	22.3	41.2	56.5
	MeJA	37.4	38.0	103.5	141.0	13.8	13.8	40.3	43.3	65.9	72.6	44.5	72.0	19.0	22.0	36.9	52.4
	NAC	39.8	38.8	100.8	138.5	13.8	13.3	39.0	42.0	62.4	74.0	43.6	71.9	19.5	21.8	35.1	51.8
AT	Vc+Ve+MeJA+Br	30.5	33.5	129.3	155.5	13.5	13.8	51.5	51.8	85.3	93.9	71.4	86.8	21.3	23.0	56.4	69.8
	Br+Tr+MeJA	31.8	34.0	126.8	152.0	13.8	13.3	48.3	48.8	83.2	92.1	68.6	84.3	20.8	22.6	54.8	67.2
	Vc+Ve	30.5	33.5	127.8	153.8	13.5	13.8	50.0	50.8	84.6	92.7	70.4	85.4	21.0	22.8	55.9	68.4
	MeJA	33.0	35.0	123.0	149.8	13.8	13.8	46.3	47.5	82.6	91.4	67.5	83.6	20.5	22.3	54.2	65.7
	NAC	34.3	36.5	119.5	149.0	13.8	13.3	45.3	46.8	81.4	90.3	66.9	83.0	20.3	22.0	53.4	65.0
Temperature		^*^	^**^	^**^	^**^	ns	ns	^**^	^**^	^**^	^**^	^**^	^**^	^**^	^*^	^**^	^**^
PGR		Ns	ns	^*^	^*^	ns	ns	^*^	^*^	^*^	^*^	^**^	^*^	^*^	ns	^*^	^*^
Temperature × PGR		ns	ns	ns	ns	ns	ns	ns	ns	ns	ns	ns	ns	ns	ns	ns	Ns
CV		10.6	10.3	6.7	6.5	7.1	5.7	5.9	6.6	5.2	5.9	4.4	4.3	4.9	7.5	8.4	8.8

^**^ and ^*^ denote significance at the 0.01 and 0.05 probability level, respectively. ns, non-significant; CV, Coefficient of variation; PGR, Plant growth regulators; HHZ, Huanghuazhan (heat tolerant), IR-64 (heat susceptible). HDT, high day temperature; HNT, high night temperature; AT, ambient temperature (control). Different PGRs viz., vitamin C (Vc), vitamin E (Ve), methyl jasmonates (MeJA), brassinosteroids (Br) and triazoles (Tr) were applied at the rate of 1.4, 6.9, 1.8, 4.0, and 0.55 ppm solution, respectively in respective treatments; NAC, nothing applied control.

The PGRs were effective in improving rice yield and its components particularly under high temperature (Tables 5, 6). The spikelet per panicle, spikelet fertility, grain filling and 1000 grain weight were not significantly affected by PGR combinations. The Vc+Ve and Vc+Ve+MeJA+Br combinations surpass all other treatments for rice yield and its related attributes. In IR-64, the number of spikelet per panicle under AT, HDT and HNT were increased by 7.97, 12.31, and 9.73%, respectively due to different PGRs combinations compared with NAC (Tables 5, 6). Likewise, spikelet fertility (14.14 and 20.05%), and grain filling (21.21 and 13.16%) were improved in IR-64 by application of PGRs under HNT and HDT, respectively. Under AT, the Vc+Ve+MeJA+Br enhanced the grain yield of IR-64 and HHZ by only 6.04 and 8.17%. Under HDT and HNT, the Vc+Ve+MeJA+Br improved the grain yield of IR-64 by 27.46 and 15.48%, and of HHZ by 62.59 and 13.64%, respectively. These results clearly indicate the effectiveness of exogenously applied PGRs in alleviating the adverse effect of HDT or HNT on grain yield of rice cultivars (Tables 5, 6).

## Discussion

Wuhan (experiment site) is known as one of the major rice growing region in China, irrigated through Yangtze River. Average temperature for rice vegetative growth period in this region in 22–24°C while optimum temperature for grain filling period is 22–28°C (Li et al., 2011). In most areas, an elevated temperature up to 35–40°C is observed during grain filling period that is too harsh for normal grain development and filling. This rise in temperature interacts with normal functioning of plants through disturbance in many physiological processes and biochemical reactions (Larkindale and Knight, 2002). Peng et al. (2004) reported that high night time temperature increase is more fatal than increase in day time temperature. Variable observations regarding the intensity and effects of high temperature were previously reported for rice physiological processes. Alghabari et al. (2014) reported that HDT was more fatal to grain set and pollen fertility in cereals while Shah et al. (2011) observed poor rice performance under HNT compared with HDT. Narayanan et al. (2014) reported similar effect of HDT and HNT on plant physiology and growth when applied at anthesis stage of crop development. Our results depicted the variable effects of HDT and HNT for different studied parameters. The HDT resulted in greater reductions in *A* and WUE (Tables 1, 2). The IR-64 was more prone to high temperature stress as apparent by the enhanced level of E, gs, and LWP, whilst HHZ showed better level of tolerance to day and night thermal changes as apparent from higher rate of *A* and WUE under stress (Tables 1, 2).

Compared with AT, high temperature considerably reduced the morpho-physiological growth of rice (Tables 1–4). Reduction in morpho-physiological growth traits have been recorded to be accountable for decrease in rice yield production under high temperature (Li et al., 2011; Narayanan et al., 2014). Under heat stress, the thylakoid membrane becomes more permeable which increases proton leakage, affects normal synthesis of adenosine triphosphate (ATP) and nicotinamide adenine dinucleotide phosphate hydrogen (NADPH), and consequently decreases the rate of *A* (Schrader et al., 2004; Narayanan et al., 2014). Loka and Oosterhuis (2010) reported that high temperature decreased *A*, sugar and starch content in rice while increased respiration and male sterility (Mohammed and Tarpley, 2009b). Guo et al. (2005) found that lower *A* under heat stress was due to reduction in leaf chlorophyll contents. Damage to electron transport chain especially at photosynthesis II or inhibition of CO_2_ fixation under heat stress may also disturb photosynthesis which is the key process for photo-assimilate production (Havaux and Tardy, 1996; Yamane et al., 1997).

Due to high rate of E generated by HDT, the lower WUE was observed under HDT than HNT (Tables 1, 2). This could also be ascribed to reduced rate of *A* under HDT. The WUE defines the amount of photosynthate accumulation or biomass production based on amount of water used during E. Higher WUE resulted in higher plant biomass and yield. In the present study, decline in WUE could also be reason for grain yield reduction under HNT. Mohammed et al. (2014) reported similar results pertaining to WUE and noted significant decline in WUE under high temperature, which was associated with higher E. Under high temperature, higher Ci compared with AT (Tables 1, 2) suggested the inability of the leaf to fix the available C due to heat stress. We observed that higher Ci did not increase *A* but gs was higher, which indicated that both rice cultivars were efficiently able to enhance influx of CO_2_ into leaves but due to high temperature induced detrimental effects on *A*, rice plants were not capable to fix that C into carbohydrates. In some earlier researches similar findings have been accounted which show that enhancement in gs or Ci did not affect *A* (Mohammed and Tarpley, 2009a).

Our study depicted slight improvement in plant height under HDT and HNT as compared with AT (Tables 3, 4). Heat-associated increment in plant height can be attributed to speedy elongation of internodes. This intermodal elongation may be linked to some internal hormones that were up-regulated under high temperature like ethylene and gibberellin (Qi et al., 2010). Our results are in consistent with the findings of Cheng et al. (2009) who reported considerable enhancement in rice plant height under HNT whereas results of some previous researchers are in contradictory with our findings who accounted HNT treatment did not significantly affect the morphological traits of rice including plant height (Mohammed and Tarpley, 2009a). High temperature reduced the leaf area and biomass accumulation in both rice cultivars as compared with AT (Tables 3, 4). Moreover, considerable decrease in leaf area guided to less *A* with associated reduction in the photosynthates assimilation in grain ultimately leading to a reduced grain yield. Mohammed and Tarpley (2014) authenticated that production area for total photosynthates was decreased due to leaf area reduction under high temperature stress, which resulted to considerable decline in grain yield. Additionally, higher night or day temperature activated photorespiration out coming in discrepancy between photosynthesis and respiration causing decline in grain yield (Venkatramanan and Singh, 2009).

Rice grain yield is dependent on number of panicles, number of spikelets per panicle, grain filling rate and total grain weight (Kim et al., 2009). High temperature particularly at night time decreased the grain numbers per spike, grain filling rate and total grain weight. Narayanan et al. (2014) also found considerable decrease in seed set, number of grains per spike and grain yield in wheat under high temperature and they attributed decreased grain number to poor seed set. Decrease in grain weight due to high temperature might be ascribed to reduced transfer of assimilates in grains and lower spikelet fertility under stress conditions (Tables 5, 6). Calderini et al. (2001) argued that reduced mobilization of stored pre-anthesis assimilates and reduced grain filling decreased the 1000-grain weight. Li et al. (2011) also reported that HNT was more harmful to grain weight in rice than HDT. Zakaria et al. (2002) reported that rate of grain growth was heat dependent and reduction in grain filling duration and dry matter accumulation led to smaller and imperfect grains. The apparent decrease in grain yield under high temperature suggested that HNT and HDT led to production of both smaller and imperfect grains. Our results also clarified pronounced differences between cultivars regarding growth and yield performance under high temperature (Tables 1–6). The HHZ performed better than IR-64 with better growth and higher grain yield under high temperature stress. These results are in confirmation with our previous reports (Fahad et al., 2015a,b, 2016), which revealed that HHZ is comparatively tolerant to high temperature compared with IR-64.

In the present work, application of PGRs was effective in alleviating the adverse effects of high temperature stress on rice. Exogenous application of PGRs considerably increased growth and yield of both rice cultivars as compared with NAC (Tables 1–6). Previously, Mohammed and Tarpley (2009b) reported that exogenous application of vitamin E, glycine betaine and salicylic acid improved rice yield and the ability of plants to tolerate high temperature stress. Better leaf gas exchange was observed when PGRs were exogenously applied as compared with NAC (Tables 1, 2). High rate of *A* in PGR-treated plants might be linked with higher WUE and lower E. In rice, application of PGR has been reported to increase leaf photosynthetic rates and decreases internal CO_2_ concentration, respiration rates and membrane injury in rice (Farooq et al., 2008a; Khan et al., 2010). We observed better Ci in rice due to application of PGRs because of higher *A* and gs (Tables 1, 2), which resulted in higher C fixation and photosynthates production. Higher 1000-grain weight in PGR-applied plants might be due to enhanced mobilization of stored pre-anthesis assimilates and higher grain filling rate. Our results depicted that the combination of Vc+Ve+MeJA+Br outperformed than all other treatments for augmenting high temperature tolerance in both rice cultivars. The highest grain yield under AT as well as high temperature stress by this PGR combination might be attributed to higher *A*, WUE, spikelet fertility and grain filling than rest of the treatments.

The increase in spikelet fertility and 1000-grain weight by PGRs application (Tables 5, 6) might be attributed to augmented mobilization of stored pre-anthesis assimilates to the grain as a result of membrane stability and/or better pollen germination (Data not shown). Although the combination of various PGRs selected in current research have never been tested earlier, however, the individual function of Vc (Guo et al., 2005; Shah et al., 2011), Ve (Munné-Bosch and Alegre, 2002), MeJA (Tani et al., 2008; Clarke et al., 2009) and Br (Li et al., 2007; Zhang et al., 2007) against abiotic stresses is well apparent.

In summary, high temperature stress severely reduced the morpho-physiological growth and yield performance of rice cultivars in both years. The HDT posed more negative effects on rice physiological traits while HNT was more lethal for grain formation and rice yield. Exogenous application of PGRs was effective in alleviating the detrimental effects of heat stress. The combination of Vc+Ve+MejA+Br was more effective than all other PGR treatments for most of studied characteristics. The highest grain yield by this PGR combination was associated with enhanced *A*, spikelet fertility and grain filling, which compensated the adversities of high temperature stress. Taken together, these results may enhance our understanding regarding adaptation and survival mechanisms of rice to high temperature and advocate the usefulness of PGRs in alleviating the adversities of high temperature on rice growth and yield. However, further studies are required test the feasibility of these PGR combinations against different abiotic stresses in rice and other crops particularly in field conditions.

## Author contributions

SF, JH initiated and designed the research, SF done the experiments, SF, SHu, SS, SHa, analyzed the data and wrote the manuscript, ZI, CW, AS, MY, WN, HA, FA, and JH revised and edited the manuscript and also provided advice on the experiments.

### Conflict of interest statement

The authors declare that the research was conducted in the absence of any commercial or financial relationships that could be construed as a potential conflict of interest.
